# Neurosyphilis Diagnosed on the Basis of Pupillary Abnormalities: A Case Report

**DOI:** 10.7759/cureus.98375

**Published:** 2025-12-03

**Authors:** Seira Hayashi, Akira Watanabe, Kie Iida, Kazusa Kuwano, Tadashi Nakano

**Affiliations:** 1 Ophthalmology, Jikei University School of Medicine, Tokyo, JPN

**Keywords:** neurosyphilis, oculomotor nerve palsy, pupillary abnormalities, syphilis, tonic pupil

## Abstract

Neurosyphilis is a condition in which *Treponema pallidum* invades the central nervous system and may occur at any stage of syphilis. Early diagnosis and treatment are crucial, as advanced disease can result in locomotor ataxia and progressive paralysis. We report a case of neurosyphilis identified on the basis of pupillary abnormalities. A man in his 20s was referred to our hospital with difficulty with near vision in the right eye, after bilateral pupillary dilation had been detected during an eye examination for contact lens prescription at another clinic. Brain MRI revealed no abnormalities, but ocular deviation and motility disturbance developed concurrently. On presentation, visual acuity was 20/20 in the right eye and 20/13 in the left eye. Pupil diameters were 7.5 mm (right) and 6.9 mm (left), with weak direct, indirect, and near reflexes. Right eyelid ptosis was present, with a levator function of 7.5 mm in the right eyelid and 10 mm in the left. The right eye showed limitations in upward, downward, and medial gazes, along with external strabismus. Blood tests for *Treponema pallidum* antibody and rapid plasma reagin were positive. Contrast-enhanced MRI demonstrated swelling and enhancement of the right oculomotor nerve. Both pupils exhibited mild constriction after 0.1% pilocarpine. The patient was treated with high-dose intravenous penicillin G and three courses of methylprednisolone pulse therapy, resulting in improvement of ocular alignment and motility and partial recovery of pupillary function. This case illustrates that neurosyphilis can present with pupillary abnormalities and oculomotor nerve involvement, even at an early stage. Neurosyphilis should be considered in the differential diagnosis of unexplained pupillary changes.

## Introduction

An increasing number of cases of co-infection with human immunodeficiency virus (HIV) and syphilis have been reported worldwide [1-3], and a rising prevalence of syphilis has also been observed in Japan [4]. Neurosyphilis is a condition in which *Treponema pallidum* invades the central nervous system and can occur at any stage of syphilis. In late neurosyphilis, tabes dorsalis, or general paresis, may develop, leading to direct damage to the central nervous system, particularly the brain parenchyma. Patients may present with memory impairment, decreased concentration, irritability, and, in the terminal stage, severe cognitive decline, personality changes, and ultimately death. Therefore, early diagnosis and treatment of neurosyphilis are of great importance.

Ocular syphilis is considered a form of neurosyphilis and can manifest at any stage of *Treponema pallidum* infection. It presents with a wide variety of nonspecific ocular findings, including chorioretinitis, retinal vasculitis, optic papillitis, and iridocyclitis, making diagnosis based solely on ocular manifestations often difficult [3]. In addition to ocular syphilis, other neurosyphilitic conditions that can cause ocular symptoms include pupillary abnormalities and ocular motility disorders. Several cases have been reported in which syphilis infection was first diagnosed following ophthalmologic evaluation for such ocular findings [4-10]. According to the report by Cheng et al., pupillary abnormalities were observed in 27.4% of patients with neurosyphilis, and 9.4% of those patients presented ocular signs as the initial symptom. Therefore, pupillary findings are important in the clinical assessment of syphilis, and it is essential for ophthalmologists to remain vigilant for syphilis in clinical practice [11].

Herein, we report a case of neurosyphilis diagnosed after an ophthalmology consultation prompted by pupillary abnormalities due to oculomotor nerve palsy and tonic pupil.

## Case presentation

A man in his 20s was referred to our hospital with complaints of difficulty with near vision in the right eye. Seven days before the presentation, he had visited a local clinic for a contact lens prescription and was noted to have bilateral pupillary dilation. Five days before the presentation, brain magnetic resonance imaging (MRI) was performed, but no abnormalities were detected. Around the same time, he developed ocular deviation, which limited his extraocular movements. He had no subjective symptoms of headache.

**Figure 1:**
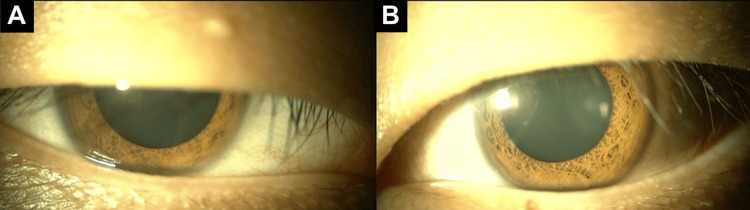
Pupillary status at the initial visit The pupil diameter measured 7.5 mm in the right eye (A) and 6.9 mm in the left eye (B), both dilated.

At his initial visit to our hospital, best-corrected visual acuity (BCVA) was 20/20 in the right eye and 20/13 in the left eye. The pupil diameter measured 7.5 mm in the right eye and 6.9 mm in the left eye, both dilated in room illumination. Light reflexes, both direct and consensual, were sluggish bilaterally, and the near response of the right pupil was markedly diminished. Right upper eyelid ptosis was also observed, with levator function reduced to 8 mm in the right eye compared to 10 mm in the left eye (Figure 1).

**Figure 2:**
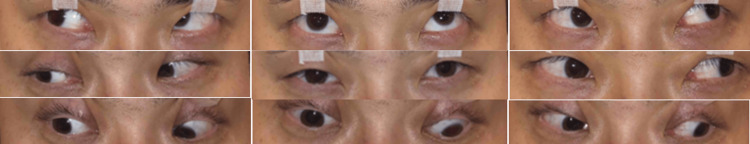
Ocular motility at the initial visit

The right eye demonstrated limitation of elevation, depression, and adduction, with 30 prism diopters of exotropia in the primary position with left-eye fixation. Extraocular movements of the left eye showed no limitation (Figure 2).

**Figure 3:**
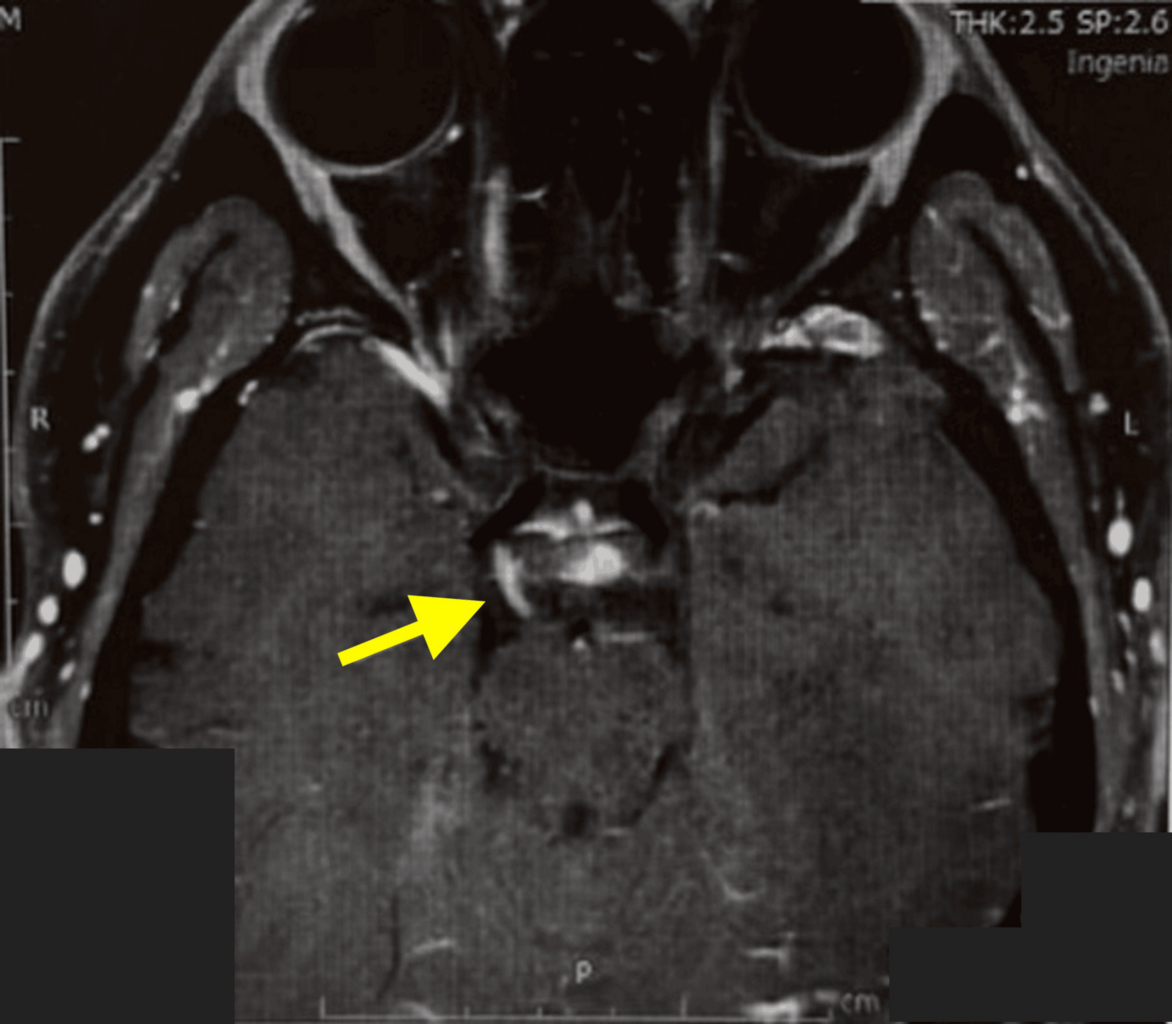
Contrast-enhanced brain MRI Brain MRI demonstrated swelling and enhancement of the right oculomotor nerve (yellow arrow).

Contrast-enhanced brain MRI demonstrated swelling and enhancement of the right oculomotor nerve. No aneurysm or other abnormal intracranial findings were identified (Figure 3).

Serologic tests were positive for both *Treponema pallidum* latex agglutination (TPLA) and rapid plasma reagin (RPR). Quantitative assays revealed a TP antibody titer of 3220.8 U/mL and an RPR of 124.8 R.U. The other laboratory findings are summarized in Table 1. A complete blood count and differential showed no evidence of immunosuppression; HIV antigen/antibody testing was negative. Cerebrospinal fluid (CSF) examination showed a protein concentration of 18 mg/dL (reference range: 15-45 mg/dL), a glucose concentration of 93 mg/dL (reference range: 50-80 mg/dL), and a cell count of 6/µL (reference range: 5/µL or less), consisting of 5 mononuclear cells/µL and 1 polymorphonuclear cell/µL. CSF syphilis testing was positive, with TP antibody 314 U/mL. The intrathecally produced *Treponema pallidum* antibody (ITPA) index, calculated as (CSF TPHA/IgG) / (serum TPHA/IgG), was 10.6 (normal < 3).

**Table 1:** Laboratory data at the initial presentation

Parameter	Result	Unit	Reference Range Lower	Reference Range Upper
Aspartate Aminotransferase (AST, GOT)	57	U/L	13	30
Alanine Aminotransferase (ALT, GPT)	147	U/L	10	42
Lactate Dehydrogenase (LD, LDH)	197	U/L	124	222
Cholinesterase (ChE)	460	U/L	240	486
Total Bilirubin (T-Bil)	0.7	mg/dL	0.4	1.5
Direct Bilirubin (D-Bil)	0.2	mg/dL	0	0.3
Indirect Bilirubin (I-Bil)	0.5	mg/dL	-	-
Alkaline Phosphatase (ALP, IFCC)	68	U/L	38	113
Leucine Aminopeptidase (LAP)	72	U/L	35	80
Gamma-Glutamyltransferase (γ-GT, γ-GTP)	77	U/L	13	64
Total Protein (TP)	7.9	g/dL	6.6	8.1
Albumin	59.7	%	55.8	66.1
α1-Globulin	3.3	%	2.9	4.9
α2-Globulin	8.5	%	7.1	11.8
β1-Globulin	6.5	%	4.7	7.2
β2-Globulin	6.3	%	3.2	6.5
γ-Globulin	15.7	%	11.1	18.8
Albumin/Globulin Ratio (A/G)	1.5	-	1.3	1.9
Creatine Kinase (CK)	132	U/L	59	248
Lactic Acid	1.6	mmol/L	0	1.8
Urea Nitrogen (UN)	14	mg/dL	8	20
Creatinine (Cr)	0.68	mg/dL	0.65	1.07
Estimated Glomerular Filtration Rate (eGFR)	122	mL/min/1.73m^2^	60	-
Uric Acid (UA)	6.8	mg/dL	3.7	7
Sodium (Na)	140	mEq/L	138	145
Potassium (K)	4.6	mEq/L	3.6	4.8
Chloride (Cl)	105	mEq/L	101	108
Total Cholesterol (TC)	195	mg/dL	142	219
Triglycerides (TG)	86	mg/dL	40	149
Fasting Blood Glucose	91	mg/dL	73	109
Free Triiodothyronine (FT3)	3.65	pg/mL	2.36	5
Free Thyroxine (FT4)	1.38	ng/dL	0.88	1.67
C-Reactive Protein (CRP)	0.39	mg/dL	-	0.14
Rheumatoid Factor (RF)	5.0>	IU/mL	0	15
Angiotensin-Converting Enzyme (ACE)	9.9	U/L	8.3	21.4
Anti-Acetylcholine Receptor Antibody (AChR Ab, RIA)	0.2>	nmol/L	0	0.2
Soluble Interleukin-2 Receptor (sIL-2R)	590	U/mL	122	496
Immunoglobulin G4 (IgG4)	27.9	mg/dL	11	121
Antinuclear Antibody (ANA, IF)	Negative	-	0	39
Anti-Thyroglobulin Antibody (Tg Ab)	15	IU/mL	0	27
Anti-Thyroid Peroxidase Antibody (TPO Ab)	15	IU/mL	0	15
TSH Receptor Stimulating Antibody (TSAb)	93	%	0	109
Herpes Simplex Virus Antibody (HSV, CF test)	4>	-	0	3
Varicella-Zoster Virus Antibody (VZV, CF test)	4>	-	0	3
Treponema Pallidum Latex Agglutination (TPLA) Test	Positive	-	-	-
Rapid Plasma Reagin (RPR) Test for Syphilis	Positive	-	-	-
White Blood Cell Count (WBC)	11.7	×10^3^/µL	3.3	8.6
Red Blood Cell Count (RBC)	5.54	×10^6^/µL	4.35	5.55
Hemoglobin Concentration (Hb)	15.5	g/dL	13.7	16.8
Hematocrit (Hct)	47.5	%	40.7	50.1
Mean Corpuscular Volume (MCV)	85.7	fL	83.6	98.2
Mean Corpuscular Hemoglobin (MCH)	28	pg	27.5	33.2
Mean Corpuscular Hemoglobin Concentration (MCHC)	32.6	g/dL	31.7	35.3
Red Cell Distribution Width - Coefficient of Variation (RDW-CV)	12.5	%	11.1	14.7
Platelet Count (PLT)	431	×10^3^/µL	158	348
Mean Platelet Volume (MPV)	9.2	fL	8.4	12.8
Platelet Distribution Width (PDW)	10	fL	8	14.5
Neutrophils	76.5	%	40.6	76.4
Lymphocytes	17.1	%	16.5	49.5
Monocytes	5.2	%	2	10
Eosinophils	0.9	%	0	8.5
Basophils	0.3	%	0	2.5
Neutrophil Count	9	×10^3^/µL	1.7	6.3
Lymphocyte Count	2	×10^3^/µL	1	3.1
Monocyte Count	0.6	×10^3^/µL	0.1	0.6
Eosinophil Count	0.1	×10^3^/µL	0	0.5
Basophil Count	0	×10^3^/µL	0	0.2
Nucleated Red Blood Cells	0	/100 WBC	0	0
Eosinophils	0.9	%	0	8.5
Basophils	0.3	%	0	2.5
Monocytes	5.2	%	2	10
Lymphocytes	17.1	%	16.5	49.5
Neutrophils	76.5	%	40.6	76.4
Erythrocyte Sedimentation Rate (ESR) at 1 hour	11	mm	2	10

Three months after treatment initiation, a low-dose pilocarpine (0.1%) test was performed: pre-instillation pupil diameters were 4 mm OD and 5 mm OS, and post-instillation, both pupils measured 3 mm, indicating cholinergic hypersensitivity.

During the course of the disease, there were no other systemic symptoms or findings due to syphilis other than the ocular findings. Based on these findings, the patient was diagnosed with right oculomotor nerve palsy and bilateral tonic pupils secondary to neurosyphilis.

Clinical course and treatment

**Figure 4:**
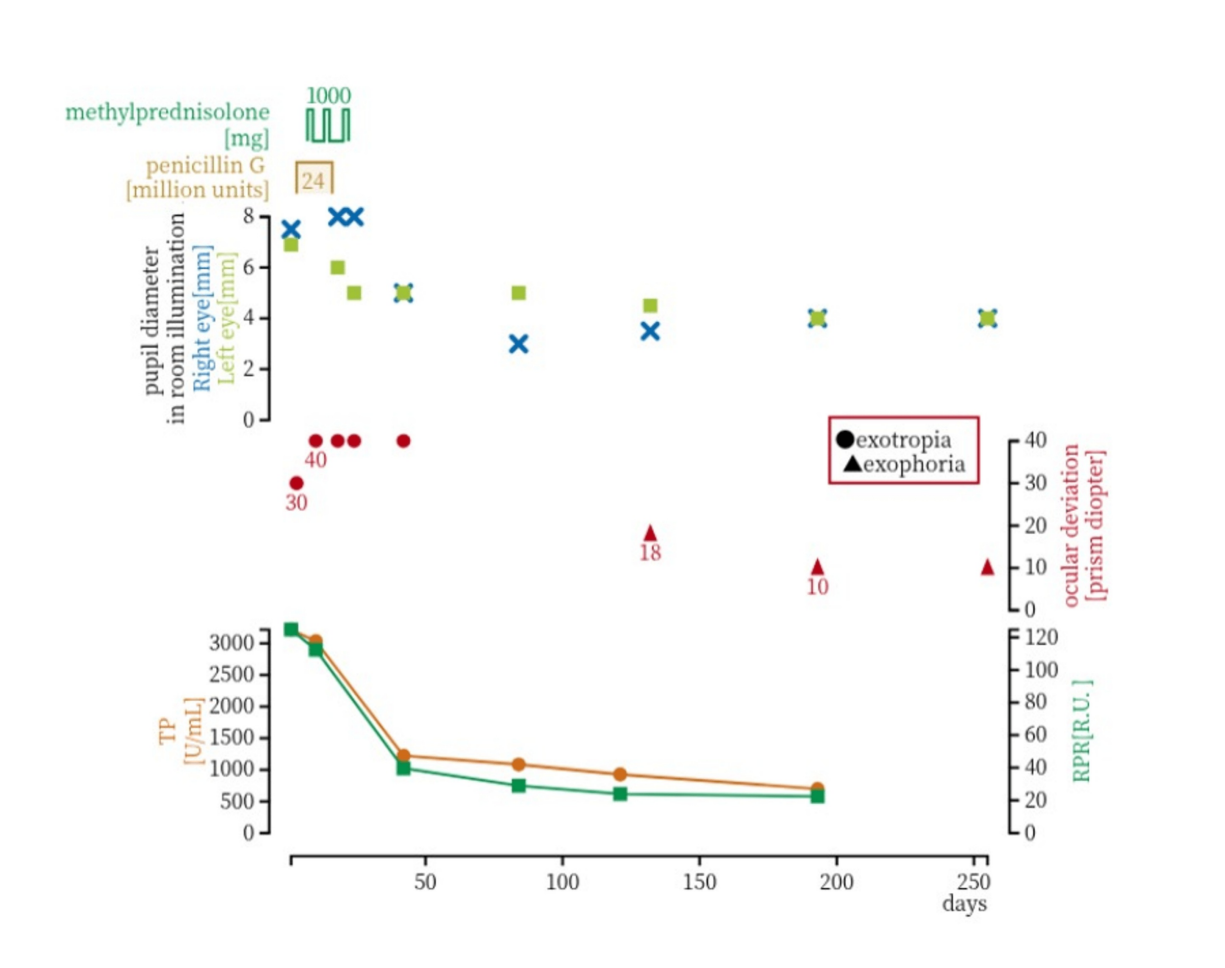
Summary of treatment and clinical course in neurosyphilis Clinical course showing the treatment regimen, changes in pupil diameter, ocular alignment, and serological test results. Penicillin G (total dose of 24 million units; days 3–16) and intravenous methylprednisolone (1,000 mg/day on days 7–9, 14–16, and 21–23) were administered. Following treatment, TP and RPR titers markedly decreased, and subsequent gradual improvement in pupil diameter and ocular alignment was observed.

The treatment course and clinical progression are presented in Figure 4. The patient was started on high-dose intravenous penicillin G potassium (40,000 units IV every four hours (six times daily) for 14 days). Four days after initiation of penicillin, high-dose intravenous methylprednisolone (IVMP; 1000 mg daily for three consecutive days) was also administered and repeated three times in total.

**Figure 5:**
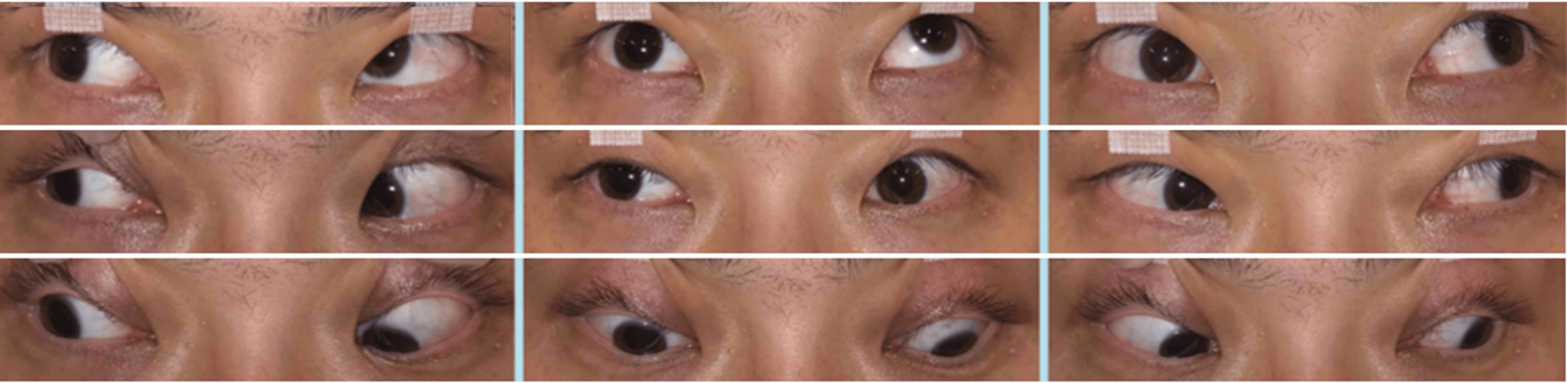
Ocular motility after two cycles of high-dose penicillin therapy combined with methylprednisolone pulse therapy Improvement of pupillary abnormalities and right ocular motility disorder remained minimal.

After completion of the 14-day penicillin G course and the second IVMP cycle, the pupil diameters were 8 mm in the right eye and 6 mm in the left eye; however, at that stage, improvement of pupillary abnormalities and right ocular motility disorder remained minimal (Figure 5).

**Figure 6:**
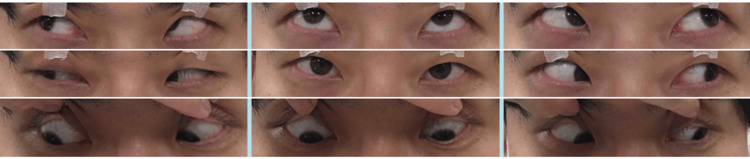
Ocular motility three months after the initial visit Gradual improvement of both ocular motility and pupillary abnormalities was observed from approximately three months after the initial visit.

Subsequently, following the third IVMP cycle and continued observation, gradual improvement in both ocular motility and pupillary abnormalities was observed approximately three months after the initial visit (Figure 6).

Four months after the first presentation, pupillary findings were as follows: in bright light, pupil diameter was 3.5 mm in the right eye and 4.5 mm in the left eye; in darkness, 5 mm in the right eye and 6 mm in the left eye. Ocular alignment measured 18 prism diopters of exophoria, and the patient reported subjective improvement of diplopia.

Eight months after the initial presentation, the direct and consensual pupillary light reflexes were present in both eyes; the right eye showed a relatively brisk response, whereas the left eye remained slightly sluggish. Although the patient reported subjective improvement compared with the pre-treatment status, he continued to experience photophobia outdoors, indicating that full recovery had not been achieved.

## Discussion

In recent years, the number of syphilis patients in Japan has increased, and neurosyphilis may occur even in the early stages of the disease [4]. Neurosyphilis is a central nervous system infection caused by *Treponema pallidum* and is classified into asymptomatic, syphilitic meningitis, meningovascular, general paresis, and tabes dorsalis forms. As the disease progresses, recovery becomes increasingly difficult, underscoring the importance of early diagnosis and treatment.

Ocular manifestations of neurosyphilis include cranial nerve palsies involving the extraocular muscles [4], autonomic dysfunction affecting the intraocular muscles, and pupillary abnormalities [5-10]. Among extraocular muscle palsies, oculomotor nerve palsy [4,10,12-14], as seen in the present case, and abducens nerve palsy have been reported [13]. However, in early neurosyphilis, the most frequently affected cranial nerves are the seventh (facial nerve), eighth (vestibulocochlear nerve), and third (oculomotor nerve), whereas abducens palsy is relatively rare [13]. In our case, right ptosis, restricted ocular motility, and mydriasis were observed, and contrast-enhanced MRI demonstrated enhancement of the oculomotor nerve itself, suggesting that neurosyphilis was the cause of the right oculomotor nerve palsy.

Similar cases have been reported in which there were no meningeal signs, no cranial nerve palsy other than oculomotor involvement, MRI showed oculomotor nerve enhancement, and intravenous methylprednisolone pulse therapy was effective [4]. These findings suggest a pathogenic mechanism of microcirculatory disturbance due to syphilitic meningeal vasculitis, which may also explain the present case. Several reports have described oculomotor nerve palsy with enhancement of the oculomotor nerve on MRI in neurosyphilis [7], whereas a non-contrast MRI can appear normal in patients presenting with diplopia who are ultimately diagnosed with neurosyphilis [11]. These observations indicate that contrast-enhanced MRI is useful for diagnosing neurosyphilitic oculomotor nerve palsy.

Pupillary abnormalities in neurosyphilis are most classically represented by the Argyll Robertson (AR) pupil [15], characterized by miosis, loss of light reflex, and preservation of near response. The lesion is thought to involve the midbrain, disrupting the pupillary light reflex pathway [5]. Tonic pupil can present with findings similar to AR pupil [5], and both unilateral [7-9] and bilateral tonic pupils associated with neurosyphilis have been reported [5,6]. The prevalence of syphilis among patients with tonic pupils has been reported to be approximately 8% [9]. Because brain MRI findings are normal in most cases of tonic pupil, imaging studies such as MRI are generally not useful for establishing the diagnosis. Instead, the diagnosis of tonic pupil relies on demonstrating hypersensitivity to dilute pilocarpine [5]. The distinction between an AR pupil and a tonic pupil lies in the hypersensitivity to dilute pilocarpine and the presence of vermiform iris movements. Because their clinical features overlap, they may be confused in practice. Tonic pupils in syphilis are thought to indicate postganglionic parasympathetic or ciliary ganglion involvement due to inflammation or ischemia [5].

In idiopathic tonic pupils, approximately 80% of cases are unilateral, and the condition occurs more commonly in women. In contrast, tonic pupils secondary to syphilis have been reported to be bilateral and predominantly affect men, as seen in the four cases described by Sakai et al. and the five cases reported by Fletcher et al. [5,9]. Therefore, clinicians should consider neurosyphilis as an important differential diagnosis when encountering bilateral tonic pupils, particularly in younger male patients without a prior history of neurological disease.

In the present case, a dilute pilocarpine test performed four months after initiation of syphilis treatment revealed hypersensitivity in both eyes. Although the right pupillary abnormality was also influenced by oculomotor nerve palsy, the findings indicated bilateral tonic pupils. To the best of our knowledge, there have been no previous reports of a case presenting with unilateral oculomotor nerve palsy and concurrent bilateral tonic pupils, making the present case the first such report.

According to the latest Centers for Disease Control and Prevention (CDC) guidelines, neurosyphilis should be treated with high-dose aqueous crystalline penicillin G, typically at a total daily dose of 18-24 million units for 10-14 days [16,17]. This regimen remains the standard of care for neurosyphilis and is widely recommended in current clinical practice. Several reports describe cases in which neurological improvement was insufficient with penicillin alone, but clinical improvement was observed when combined with steroid pulse therapy [13,14]. Accordingly, systemic corticosteroids are often administered as adjunctive therapy in otosyphilis and ocular syphilis; however, their therapeutic efficacy has not yet been firmly established [17]. In the present case, multiple cranial neuropathies were initially suspected, and steroid pulse therapy was therefore added four days after initiation of penicillin. Although improvements in pupil diameter, ocular alignment, and ocular motility were observed in this case, these changes occurred gradually following the decline in syphilis serological titers rather than immediately after the steroid pulses. Therefore, whether the clinical improvement was attributable to corticosteroid therapy remains inconclusive. The indications and optimal timing of steroid pulse therapy in neurosyphilis are not yet established, highlighting the need for prospective studies and the accumulation of additional cases.

## Conclusions

We report a rare case of unilateral oculomotor nerve palsy with concurrent bilateral tonic pupils caused by neurosyphilis. The patient was treated with high-dose intravenous penicillin G and three courses of intravenous methylprednisolone pulse therapy, resulting in improvement of ocular alignment and motility and partial recovery of pupillary function. Contrast-enhanced MRI was useful for diagnosing neurosyphilitic oculomotor nerve palsy, and the low-dose pilocarpine test was effective in confirming tonic pupils. It is important to consider neurosyphilis as an underlying cause of pupillary abnormalities. In particular, because idiopathic bilateral tonic pupils are uncommon, neurosyphilis should be suspected in young male patients presenting with bilateral tonic pupils.

## References

[1] Savage EJ, Marsh K, Duffell S, Ison CA, Zaman A, Hughes G (2012). Rapid increase in gonorrhea and syphilis diagnoses in England in 2011. Euro Surveill.

[2] Fonollosa A, Martinez-Indart L, Artaraz J (2016). Clinical manifestations and outcomes of syphilis-associated uveitis in Northern Spain. Ocul Immunol Inflamm.

[3] Li SY, Birnbaum AD, Tessler HH, Goldstein DA (2011). Posterior syphilitic uveitis: clinical characteristics, co-infection with HIV, response to treatment. Jpn J Ophthalmol.

[4] Soeda S, Onoue H, Shinmura Y, Ebihara S, Suzuki T, Akaiwa Y, Miyamoto T (2022). A case of meningovascular syphilis presenting with bilateral oculomotor nerve palsy, cerebral aneurysm, and cerebral hemorrhage (Article in Japanese). Rinsho Shinkeigaku.

[5] Sakai T, Shikishima K, Mizobuchi T, Yoshida M, Kitahara K (2003). Bilateral tonic pupils associated with neurosyphilis. Jpn J Ophthalmol.

[6] Yasaki S, Ohshima J, Yonekura J, Takahashi Y, Someya K (1992). A case of early syphilis presenting general paresis-like symptoms and bilateral tonic pupils (Article in Japanese). Rinsho Shinkeigaku.

[7] Camoriano GD, Kassab J, Suchak A, Gimbel HV (2011). Neurosyphilis masquerading as an acute Adie's tonic pupil: report of a case. Case Rep Ophthalmol.

[8] Englestein ES, Ruderman MI, Troiano RA, Digiovanni VJ (1986). Dilated tonic pupils in neurosyphilis. J Neurol Neurosurg Psychiatry.

[9] Fletcher WA, Sharpe JA (1986). Tonic pupils in neurosyphilis. Neurology.

[10] Wang X, Mu P, Zhang W, Liu Y (2021). Case report: not all neurological symptoms respond well to penicillin in patients with neurosyphilis. Front Neurol.

[11] Cheng H, Zhu H, Shen G, Cheng Y, Gong J, Deng J (2024). Ocular findings in neurosyphilis: a retrospective study from 2012 to 2022. Front Neurol.

[12] Antaki F, Bachour K, Trottier M, Létourneau-Guillon L, Rouleau J (2021). Neurosyphilis masquerading as oculomotor nerve palsy in a healthy middle-aged man: case report and review of the literature. IDCases.

[13] Shigemi J, Sato S, Hasegawa S, Shibayama H, Maki H, Ouchi T (1995). A case of meningo-vascular type neurosyphilis presenting oculomotor nerve paralysis with interesting MRI findings (Article in Japanese). Nihon Naika Gakkai Zasshi.

[14] Komamura H, Nakamura T, Kobayashi J, Harada R, Endo K, Ogura M, Higuchi J (2019). Early neurosyphilis presenting with multiple cranial nerve palsies: a case report of management by combined penicillin-corticosteroid treatment. J Infect Chemother.

[15] Robertson DA (1869). Four cases of spinal myosis; with remarks on the action of light on the pupil. Edinb Med J.

[16] Jay CA (2006). Treatment of neurosyphilis. Curr Treat Options Neurol.

[17] Workowski KA, Bachmann LH, Chan PA (2021). Sexually transmitted infections treatment guidelines, 2021. MMWR Recomm Rep.

